# Insight into Liver lncRNA and mRNA Profiling at Four Developmental Stages in Ningxiang Pig

**DOI:** 10.3390/biology10040310

**Published:** 2021-04-08

**Authors:** Yan Gong, Yuebo Zhang, Biao Li, Yu Xiao, Qinghua Zeng, Kang Xu, Yehui Duan, Jianhua He, Haiming Ma

**Affiliations:** 1 College of Animal Science and Technology, Hunan Agricultural University, Changsha 410128, China; 13910008175@139.com (Y.G.); ybzhangfd@126.com (Y.Z.); 18874028597@163.com (B.L.); xiaoyu1030189228@126.com (Y.X.); zhouwei_2005@126.com (Q.Z.); jianhuahy@hunau.net (J.H.); 2 Ningxiang Pig Farm of Dalong Livestock Technology Co. Ltd., Ningxiang 410600, China; 3 Laboratory of Animal Nutritional Physiology and Metabolic Process, Key Laboratory of Agroecological Processes in Subtropical Region, Institute of Subtropical Agriculture, Chinese Academy of Science, Changsha 410125, China; xukang2020@isa.ac.cn (K.X.); duanyehui@isa.ac.cn (Y.D.)

**Keywords:** Ningxiang pig, lncRNAs, mRNAs, STEM, liver development

## Abstract

**Simple Summary:**

The current study reveals the expression profiles and functional networks on messenger RNAs (mRNAs) and long non-coding RNAs (lncRNAs) in the liver of Ningxiang piglets across four developmental stages (30, 90, 150, and 210 days after birth). Differentially expressed mRNAs (DEmRNAs) were upregulated at 30 days; however, most differentially expressed lncRNAs (DElncRNAs) were downregulated at 210 days. A complex interaction between mRNAs and lncRNAs was identified by Short Time-series Expression Miner (STEM) analysis and weighted gene co-expression network analysis (WGCNA), indicating that lncRNAs may be a critical regulatory element in mRNAs. STEM was used to identify significant temporal expression profiles and the genes associated with them and to compare the behavior of these genes across multiple conditions. WGCNA was used to study the biological networks based on pairwise correlations between variables. One particular mRNA profile 4 contained *CAV1*, *PACSIN2*, and *CDC42*, which are the target genes of lncRNAs in the same profile, suggesting the possible regulatory relationship between lncRNAs and mRNAs.

**Abstract:**

Ningxiang pigs, a fat-type pig, are native to Ningxiang County in Hunan Province, with thousands of years of breeding history. This study aims to explore the expression profiles and functional networks on messenger RNAs (mRNAs) and long non-coding RNAs (lncRNAs) in the liver. Liver tissue of Ningxiang piglets was collected at 30, 90, 150, and 210 days after birth (four development stages), and the mRNA and lncRNA expression was profiled. Compared to mRNA and lncRNA expression profiles, most differentially expressed mRNAs (DEmRNAs) were upregulated at 30 days; however, most DElncRNAs were downregulated at 210 days. Via Short Time-series Expression Miner (STEM) analysis and weighted gene co-expression network analysis (WGCNA), a complex interaction between mRNAs and lncRNAs was identified, indicating that lncRNAs may be a critical regulatory element for mRNAs. One module of genes in particular (module profile 4) was related to fibril organization, vasculogenesis, GTPase activator activity, and regulation of kinase activity. The mRNAs and lncRNAs in module profile 4 had a similar pattern of expression, indicating that they have functional and regulatory relationships. Only *CAV1*, *PACSIN2*, and *CDC42* in the particular mRNA profile 4 were the target genes of lncRNAs in that profile, which shows the possible regulatory relationship between lncRNAs and mRNAs. The expression of these genes and lncRNAs in profile 4 was the highest at 30 days, and it is believed that these RNAs may play a critical role during the suckling period in order to meet the dietary requirements of piglets. In the lncRNA–mRNA co-expression network, the identified gene hubs and associated lncRNAs were shown to be involved in saccharide, lipid, and glucose metabolism, which may play an important role in the development and health of the liver. This result will lead to further investigation of liver lncRNA functions at various stages of development in Ningxiang pigs.

## 1. Introduction

There are about 20,000 protein-coding genes in the human genome, accounting for less than 2% of the whole genome [1]. Those sequences that do not encode proteins were once thought to be "junk sequences" or “noise” [2]. With the rapid development of high-throughput sequencing technologies, there has been more focus on the study of genome sequencing analysis [3]. The long non-coding RNA (lncRNAs), a group of RNA molecules with a transcript length of more than 200 bp, are structurally like mRNAs, but cannot encode proteins [4]. Recent research has shown that lncRNAs can regulate gene expression as key regulatory molecules at the transcriptional and post-transcriptional level and play a significant biological role in mammalian physiological and pathological processes [4,5]. As more lncRNAs are identified in humans and mammals, their regulatory relationship with corresponding potential target genes remains unclear, especially concerning the effects on fatty acid biosynthesis, transport, and metabolism.

Ningxiang pigs, a fat-type pig, are native to Ningxiang County in Hunan Province, with thousands of years of breeding history. In the long-term breeding process, Ningxiang pigs, one of the four famous pig species in China, are popular for their advantages, including tender and succulent meat, unique flavor, and high-quality unsaturated fatty acids, as compared to other local pig species. Ningxiang pig is an important national livestock resource and was approved by the Ministry of Agriculture in 2010 as a geographical representation of Chinese agricultural products [6]. In the meantime, scientific institutes established a new experimental miniature pig line using Ningxiang pigs, since miniature pigs are very close to humans in terms of anatomy, physiology, and disease [7]. In addition, a recent study suggested that monounsaturated and polyunsaturated fatty acids account for 43.10% and 12.82%, respectively, and arachidonic acid (AA; C20:4n6) and docosahexaenoic acid (DHA; C22:6n3) account for 2.29% and 0.14% of total fatty acids in the longissimus dorsi muscle of Ningxiang pig [8,9]. However, the AA and DHA content was 17.02% and 0.88%, respectively, in the liver, which was substantially higher than the muscle tissue, suggesting that the liver is an essential organ in the process of lipid biosynthesis and metabolism. Hence, it is interesting to study the regulation and control of the molecular mechanism of lipid metabolism in the liver. In recent years, the regulatory role of lncRNA in the transcription process has received great attention. Therefore, we studied the regulatory effect of lncRNA on the mRNA of Ningxiang pigs at various developmental stages, paying particular attention to the regulation of lipid metabolism.

The liver, the primary digestive organ in mammals, is involved in the metabolism of amino acids, carbohydrates, lipids, and other substances by secreting bile, digestive enzymes, and hormones. The development and functions of the liver take place from birth. Through the development and maturation of all kinds of functions, the liver plays an essential role in digestion and metabolism [10,11]. This study aims to investigate the expression profiles of lncRNA and mRNA in the liver of Ningxiang pigs in different development stages in order to determine which genes are crucial in the liver in terms of lipid biosynthesis and metabolism, and elucidate the differential expression of lncRNAs at four development stages. The selected four developmental stages all represent four important physiological nodes in the development of Ningxiang pigs. The 30-day age represents the piglet stage, the 90-day age represents the nursery pig stage, the 150-day age represents the early fattening stage, and the 210-day age represents the late fattening stage. 

In this research, we used high-throughput sequencing technology to systematically identify the lncRNAs and mRNAs of the liver at four developmental stages (30, 90, 150, and 210 days after birth). Interestingly, 30 days after birth was a significant period for lipid biosynthesis in the liver. STEM and WGCNA showed that mRNAs and lncRNAs were enriched with lipid, saccharide, and protein metabolism, and other biological processes, and they may play an important role in liver development, health, and tissue repair. In the meantime, the potential regulatory relationship between lncRNAs and mRNAs has been further confirmed. The results from this study can provide references for further study of the function and mechanisms of lncRNAs in the liver and may also provide an important reference for the research of other Chinese pig breeds. The findings from this study provide molecular basis for subsequent research on Ningxiang pigs, such as the next lncRNA function study to provide candidate genes, and at the same time understand the development characteristic of Ningxiang pigs in actual production and provide an important reference for the research on nutrition and feeding management of Ningxiang pigs. 

## 2. Materials and Methods

### 2.1. Animals and Sample Collection

A Ningxiang boar was mated with 4 Ningxiang sows to obtain half-sibling piglets. A total of 12 tails of half-sibling Ningxiang piglets were provided by the Ningxiang original breeding pig farm of Hunan Chuweixiang Agriculture and Animal Husbandry Co. Ltd. (Ningxiang, Hunan, China). Twelve healthy male full-sibling piglets with similar weights were randomly chosen to be slaughtered to collect liver samples at 4 development stages (30, 90, 150, and 210 days after birth), and 3 tails were randomly chosen for each stage. All experimental pigs were fed, reared and managed in the same manner and condition from birth. During the liver samples collection, all the liver tissue samples were collected from the same site in each animal at each collection time-point. The collected liver samples were immediately placed in liquid nitrogen, and then transferred to a freezer at −80 °C for RNA extraction. All animal experiments in this study were approved by the Institutional Animal Care and Use Committee of Hunan Agricultural University, Changsha, Hunan Province, China under approval number 2013-06. 

### 2.2. RNA Isolation, Library Construction, and RNA-seq

Total RNA from liver was isolated by TRIzol Reagent (TaKaRa Bio Inc, Dalian, China), following the manufacturer’s procedure. The isolated RNAs were treated with RNase-free DNase to eliminate excess DNA. The quality of isolated RNAs was assessed by a Nanodrop 2000 (Thermo Fisher Scientific, Waltham, MA, USA) and 1% agarose gel electrophoresis. The qualified RNAs were kept in a freezer at −80 °C until use. An RNA-seq transcriptome strand library was prepared with a TruSeqTM stranded total RNA Kit from Illumina (Illumina, San Diego, CA, USA) using 5 μg of total RNA. Ribosomal RNA (rRNA) depletion instead of poly(A) purification was performed by a Ribo-Zero Magnetic kit and then fragmented by fragmentation buffer. The first-stranded cDNA was synthesized with random hexamer primers. The RNA template was removed to synthesize a replacement strand, incorporating dUTP instead of dTTP to produce double-strand cDNA (dscDNA). AMPure XP beads were used to isolate the dscDNA from the second strand reaction mix. A single A nucleotide was added to the 3’ ends of these blunt fragments to prevent them from ligating to each other during the adaptor ligation reaction. Finally, multiple indexing adapters were ligated to the ends of the dscDNAs. Libraries were size selected for cDNA target fragments with 200–300 bp on 2% Low Range Ultra Agarose, followed by PCR amplified using Phusion DNA polymerase (NEB, MA, USA) for 15 PCR cycles. After quantification by TBS380, a paired-end RNA-seq sequencing library was sequenced with the Illumina HiSeqxten (2 × 150 bp read length). 

### 2.3. Identification and Classification of LncRNAs

Since the original sequencing data will contain sequencing adapter sequences, low-quality reads, sequences with a higher N rate, and sequences with too short length will seriously affect the quality of subsequent analysis. To ensure the quality and accuracy of subsequent biological information analysis, the original sequencing data is first filtered to obtain high quality clean data. The SeqPrep (https://github.com/jstjohn/SeqPrep, accessed on 5 October 2020) and Sickle (https://github.com/najoshi/sickle, accessed on 10 October 2020) was used to assess and trim the sequencing read quality. The linker sequence in the reads, and the read with no inserted fragments due to self-connection of the linker, were removed; low-quality (quality value less than 20) at the end of the 3’ end sequence was trimmed off; the quality value less than 10 in the remaining sequence was removed; the reads with more than 10% of N was removed; the adapter and sequence whose length is less than 20 bp after quality trimming was discarded. The base content distribution assessment is used to detect the presence or absence of AT and GC separation. We mapped the sequence by using TopHat2 [12]. The reads were aligned against the genome (the accession number of the third-generation whole genome sequencing data of the Ningxiang pig in NCBI is PPJNA531381, and the draft genome of Ningxiang pigs that were in the confidential stage and were unpublished; we have included the transcriptome GTF file in the Supplementary Materials). Reads were split into smaller segments, which were then aligned to the genome. The segment mappings were used to find potential splice sites where the distance between the mapped positions of the left and right segments were longer than the length of the middle part of a read. The sequences flanking a splice site were concatenated and segments were aligned. The mapped segments against genome and flanking sequence were gathered to produce whole read alignments. The genome mapped reads with alignments were extended a few bases into introns and realigned to exons instead. For the preliminary screening of lncRNAs, the cuffcompare program in the Cufflinks suite was used to screen the intergenic, intronic, and anti-sense lncRNAs. The screening criteria were transcripts with fragment counts ≤ 3, transcripts shorter than 200 nt, open reading frame (ORF) longer than 300 nt, and an exon number < 2. Transcripts with fragment count ≤ 3, transcripts shorter than 200 nt, ORF longer than 300 nt, and exon number < 2 were eliminated. After preliminary screening, advance screening was carried out to screen the coding potential of lncRNA. The Coding Potential Calculator (CPC), Coding-Non-Coding Index (CNCI), and Coding Potential Assessment Tool (CPAT) were used to filter transcripts with coding potential. The remaining transcripts with identified protein domains were excluded by Pfam Scan under Pfam HMM. The remaining transcripts were considered to be reliably expressed as lncRNAs. The expression level of each lncRNA was determined using the fragments per kilobase of exon per million mapped reads (FRKM) method. The significantly differently expressed (DE) lncRNAs were extracted with |log2FC| > 1, false discovery rate (FDR) < 0.05 by edgeR. The 30-day vs. 90-day, 30-day vs. 150-day, 30-day vs. 210-day, 90-day vs. 150-day, 90-day vs. 210-day, and 150-day vs. 210-day groups were undertaken to compare for the differentially expressed (DE) lncRNAs. 

### 2.4. Differential Expression Analysis and Functional Enrichment 

The adapter sequences were removed from the raw sequencing reads prior to alignment (as not removing, it will reduce the read mapping percentage). The expression level of each transcript was determined based on fragments per kilobase of exon per million mapped reads (FRKM) for the identification of differential expression genes (DEGs) among the samples tested. RSEM (http://deweylab.biostat.wisc.edu/rsem/, accessed on 8 November 2020) was used to measure gene abundance. The 30-day vs. 90-day, 30-day vs. 150-day, 30-day vs. 210-day, 90-day vs. 150-day, 90-day vs. 210-day, and 150-day vs. 210-day groups were undertaken to compare the differential expression. Empirical Analysis of Digital Gene Expression in R (edgeR) software in the R statistical package was used for the analysis of the differential expression. In addition, a functional enrichment analysis, including Gene Ontology (GO) and Kyoto Encyclopedia of Genes and Genomes (KEGG) was performed to determine which DEGs were significantly enriched in GO terms and metabolic pathways at a Bonferroni-corrected *p*-value ≤ 0.05 compared to the whole transcriptome background. The GO functional enrichment and KEGG pathway analysis were carried out by Goatools and KOBAS. 

### 2.5. Time-Series Analysis

The Short Time-series Expression Miner (STEM) clustering algorithm was used to classify protein coding genes and lncRNA expression profiles in order to explore the relationship between temporal gene expression patterns and the liver during the four stages of piglet development [13]. The novel clustering method that STEM implements first defines a set of distinct and representative model temporal expression profiles independent of the data. These model profiles correspond to possible profiles of gene expression changes over time. The model profiles started at 0, and then between two time points would hold steady, or increase or decrease an integral number of time units up to a parameter value. The number of genes assigned to each model profile is the computed value. The number of genes expected to be assigned to a profile was estimated by randomly permuting the original time point values, renormalizing the gene expression values, then assigning genes to the closest-matching model profiles, and repeating for a large number of permutations. The true order of time points was used to test the standard hypothesis and estimate the *p*-value by the number of genes from the model profile and the number of assigned genes (adjusted *p*-value ≤ 0.05 by Bonferroni correction). Model profiles with color (except white) indicate that the temporal trends of mRNAs and lncRNAs were statistically significant. Profiles that had the same color were grouped into the same cluster.

### 2.6. Co-Expression Networks

The mRNA–lncRNA co-expression networks were developed through the developmental phases of the weighted correlation network analysis (WGCNA) package of R software [14]. After eliminating samples with outliers, we determined the Pearson’s correlation coefficient between any two genes in the gene set and constructed a matrix of correlation coefficients. The sample clustering of WGCNA analysis is shown in Figure S1. In order to construct the adjacency matrix, the right threshold (β value) was selected to measure the weighted power exponent of the correlation coefficient matrix. On this basis, a topological overlap matrix (TOM) was created and applied to the connections between genes. The gene modules were divided preliminarily by hierarchical clustering analysis in order to obtain eigengenes according to related traits. Based on the similarity of eigengenes, the modules were merged, resulting in the final modules for further analysis [15].

### 2.7. Quantitative Real-Time PCR Analysis

Total RNA was extracted using an Animal Total RNA Kit (Tiangen, Beijing, China) and treated with ribonuclease R to validate the identified lncRNAs in Ningxiang pig. cDNA was synthesized by reverse transcription using the Revert Aid First Strand cDNA Synthesis Kit (Thermo Scientific, Waltham, MA, USA). The transcription levels of MSTRG.1053.3, MSTRG.11451.1, MSTRG.19861.1, MSTRG.8339.1, and MSTRG.1054.2 were validated by Quanstudio 6 Flex (Applied Biosystems, Foster City, CA, USA). The forward and reverse primers for gene quantification are listed in Table 1. RT-qPCR was performed in a 96-well plate, with each well containing 20 μL of mixture, including 10 μL of SYBR Premix Ex Taqm II (TaKaRa, Dalian, China), 0.4 μL (10 μM) of forward and reverse primer, 2 μL of cDNA template, and 7.2 μL of diethyl pyrocarbonate (DEPC) water. The RT-qPCR running conditions were set as follows: 95 °C for 5 min (pre-denaturation) and 40 cycles of amplification (95 °C for 15 s, 59 °C for 40 s, and 72 °C for 20 s). The gene validation for each time point was performed in triplicate. The expression level of each validated gene for each time point was calculated by the 2^−ΔΔCt^ method. 

## 3. Results

### 3.1. Identification and Classification of lncRNAs in Ningxiang Pig Liver

**Figure 1:**
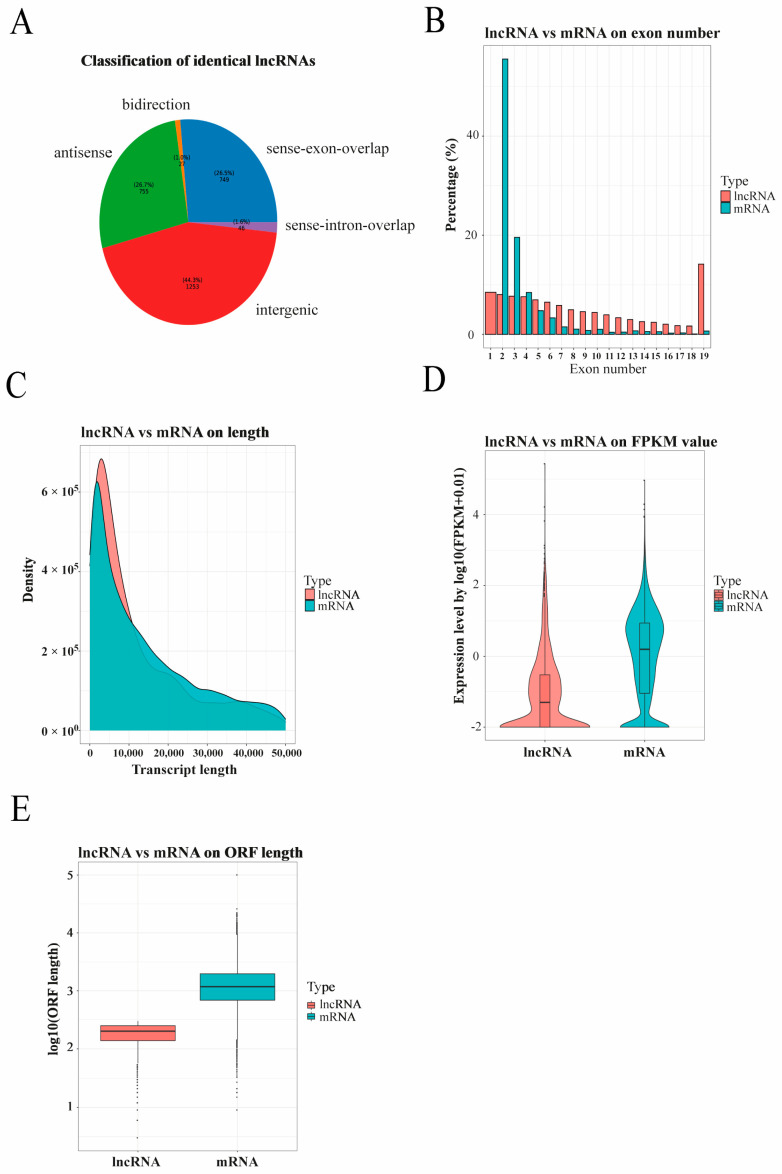
Genomic characterization and (**A**) classification of lncRNAs. lncRNA and mRNA transcripts were compared by (**B**) exon number, (**C**) length, (**D**) fragments per kilobase of exon per million mapped reads (FRKM) value, and (**E**) open reading frame (ORF) length.

After lncRNA and mRNA analysis of Ningxiang pig liver at 30, 90, 150, and 210 days after birth, 333,589,902, 328,613,762, 266,368,076, and 306,539,556 clean reads were obtained with more than 95.10% of Q30, respectively (Table 2). These clean reads were aligned to the Ningxiang pig reference genome from 92.55 to 94.73% (Table 3). A total of 2830 novel lncRNAs were identified, and these were divided into five types: intergenic (44.3%), antisense (26.7%), bidirectional (1.0%), sense exon overlapping (26.5%), and sense intron overlapping lncRNAs (1.6%) (Figure 1A and Table S1). The majority of lncRNAs contained two exons, followed by three exons, and were lower and shorter than mRNAs in terms of the expression level and length, which is consistent with previous results in humans and mammals (Figure 1B–E) [16].

**Figure 2:**
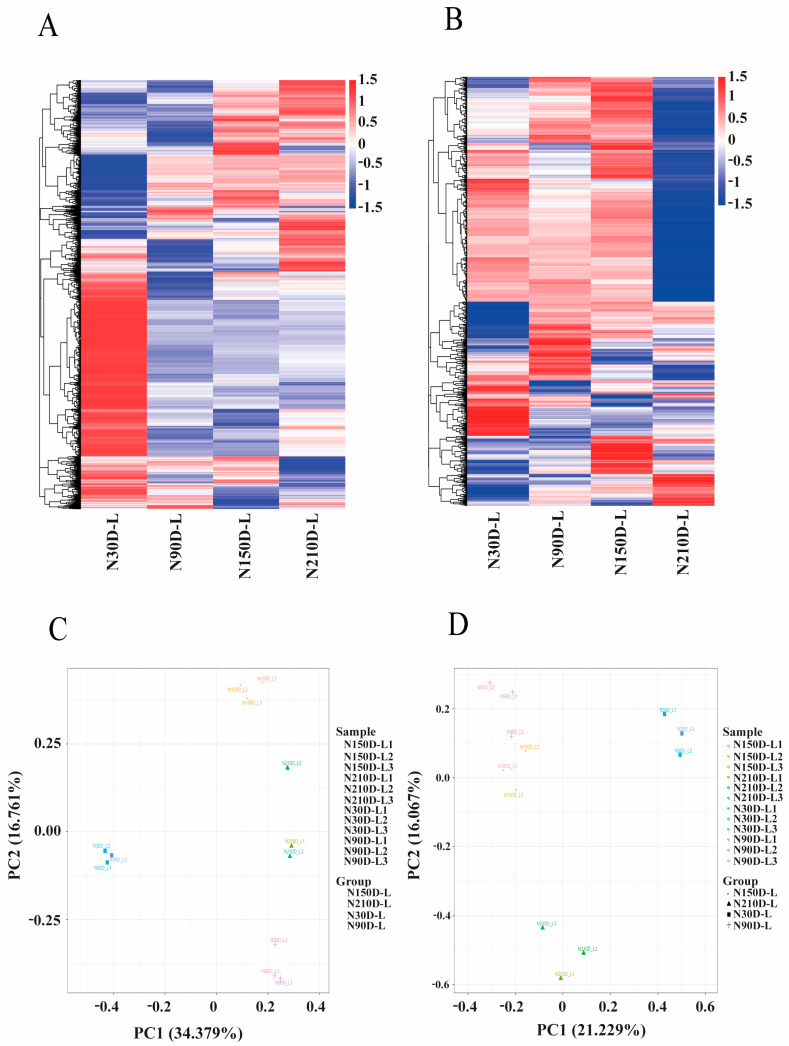
Heat maps of differentially expressed (**A**) mRNAs and (**B**) lncRNAs and their expression modes. Principal component analysis (PCA) of (**C**) lncRNAs and (**D**) mRNAs in 12 samples of liver at four stages of development.

According to the expression profiles of mRNAs, we found that the samples could be distinguished among the four development stages. Surprisingly, the 90-, 150-, and 210-day-old pigs had some similarities in the expression profiles, but the 30-day-old pigs showed the biggest differences from the other three age groups (Figure 2A). At 210 days after birth, most of the differential lncRNAs were downregulated, indicating that regulation of lncRNAs at this stage was comparatively weaker than at other stages of development (Figure 2B). All differential expressed mRNAs were compatible with the principal component analysis (PCA) study (Figure 2C). As a result, it was proposed that 30 days after birth may be the most significant development period for liver compared to the other stages. All differential expressed lncRNAs were consistent with the PCA study (Figure 2D). The PCA use expression profiling analysis, including mRNA and lncRNA. However, the WGCNA clustering uses an expression profile with the sequence set of lncRNA + mRNA, which was filtered according to the size of the expression and the coefficient of variation. Besides, the mRNA PCA uses mRNA sequences while lncRNA PCA uses lncRNA sequences. However, the WGCNA clustering uses mRNAs and the lncRNAs sequence set (which is equivalent to the sum of both mRNAs and lncRNAs).

### 3.2. Identification of Differentially Expressed Protein-Coding Genes and lncRNAs

**Figure 3:**
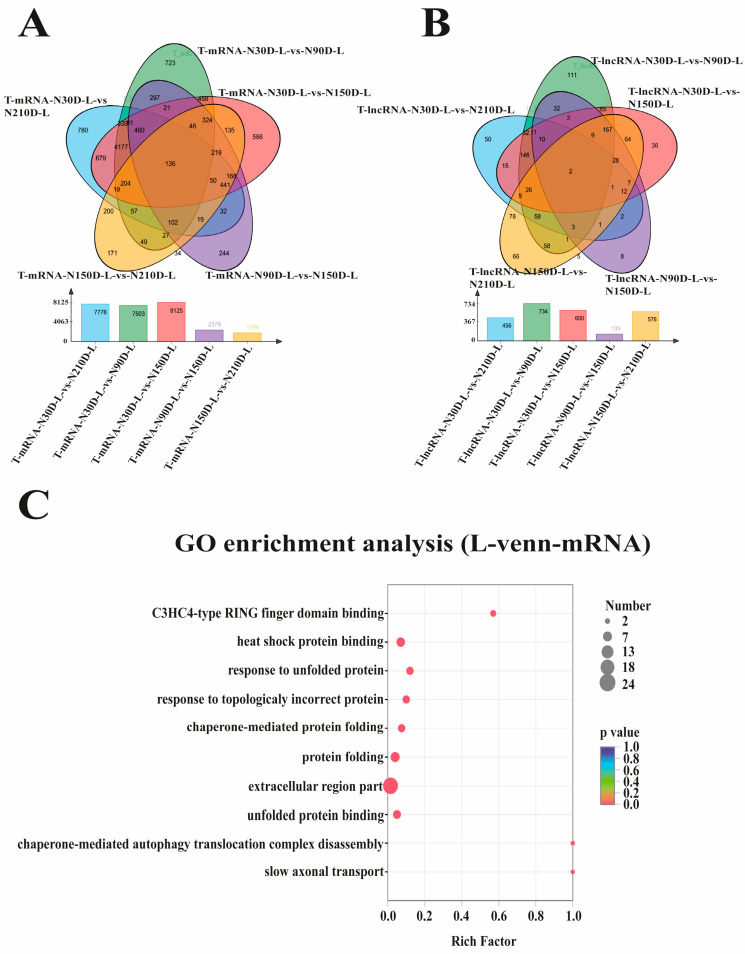
Venn diagram of common (**A**) DEmRNAs and (**B**) DElncRNAs during four stages by three closed groups and two consecutive groups, and distribution diagram of enriched Gene Ontology (GO) functions of (**C**) common DEmRNAs.

A total of 11,264 differentially expressed mRNAs (DEmRNAs) (Table 4 and Table S2) and 1158 differentially expressed lncRNAs (DElncRNAs) were identified (Table 5 and Table S3). In the DEmRNAs, the greatest variations happened at 30 days, as compared to 90, 150, and 210 days. In the DElncRNA section, the 30 day vs. 90 day stage showed the largest amount of differential expression compared to other groups. This suggests that 30 days after birth may be an important stage in liver development. To explore common DEmRNAs and DElncRNAs during the four stages of development, three closed groups (30 vs. 90 d, 30 vs. 150 d, and 30 vs. 210 d) and two consecutive groups (90 vs. 150 d and 150 vs. 210 d) were designed in order to construct Venn diagrams. The result revealed that 136 DEmRNAs and 2 DElncRNAs were differentially expressed in all four stages of development (Figure 3A,B). Among the common mRNAs, we discovered that *ACSL3*, *CES1*, *CYP2C42*, *CYP4A24*, and *PLIN4* were associated with fatty acid metabolism, indicating that it is an important part of liver development (Table S4). Enrichment analysis was performed, and the result revealed that these common DEmRNAs were significantly enriched for the extracellular region part and protein folding (Figure 3C and Table S5). The common DElncRNAs included MSTRG.31876.3 and MSTRG.33238.1, of which the latter was expected to have several target genes, such as *CDHR5*, *CYP4A24*, *HSD17B3*, and *TICAM1*. Surprisingly, these target genes were only part of the common DEmRNAs, suggesting that this lncRNA may play an important role in liver development.

### 3.3. Time-Series Analysis of Protein-Coding Genes and LncRNAs

**Figure 4:**
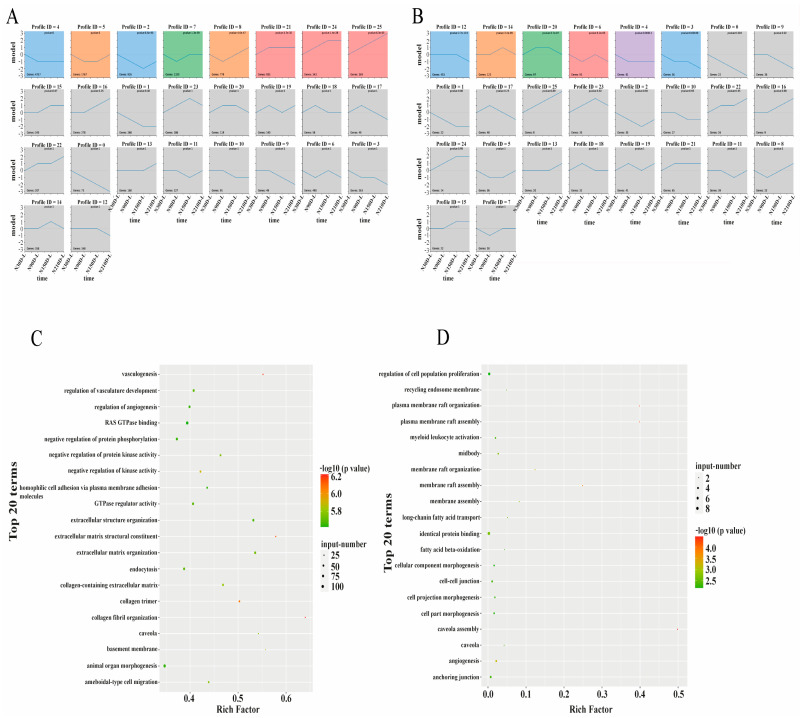
Short Time-series Expression Miner identified temporal expression profiles of (**A**) mRNAs and (**B**) lncRNAs. Top panel shows module number. Numbers at top right corner of panels represent the *p*-value and numbers at the bottom left indicate lncRNAs or mRNAs in each profile module. Profiles with same color are grouped in the same cluster. Distribution diagram of Gene Ontology functions of (**C**) mRNAs and (**D**) lncRNAs in STEM analysis.

The analysis results show that some of the protein coding genes and lncRNAs were classified into four and five cluster profiles, containing eight and six enriched model profiles, respectively. Gradual increases or decreases were distributed in mRNAs profiles 4, 21, and 24, while biphasic responding expression patterns happened in module profiles 2, 5, 7, and 8 (Figure 4A). Module profiles 6, 14, and 20 of lncRNAs showed biphasic responding expression patterns, while module profiles 3, 4, and 12 of lncRNAs showed a gradual decrease (Figure 4B). However, there were similar expression patterns in model profile 4 of protein coding genes and lncRNAs, which may suggest high correlation during different stages. Depending on the degree of significance and enrichment of the gene, GO analysis of module profile 4 showed that the genes were mainly enriched for fibril organization, vasculogenesis, GTPase activator activity, and kinase activity regulation (Figure 4C and Table S6). Meanwhile, lncRNAs were mainly enriched with long-chain fatty acids, membrane rafts, angiogenesis, and identical protein binding, and MSTRG.34993.2 was predicted to regulate *CPT1B* targeting (Figure 4D and Table S7). Protein coding genes such as *CAV1*, *PACSIN2*, and *Cdc42* in mRNA profile 4 were precisely predicted as target lncRNAs genes in lncRNA profile 4, further verifying the possible regulatory relationship between lncRNAs and mRNAs. In addition, 341 and 82 KEGG pathways of mRNAs and lncRNAs were enriched in profile 4, respectively. mRNAs are involved in the phospholipase-D, PI3K-Akt, and AGE-RAGE signaling pathways and the ECM–receptor interaction (Table S8). lncRNAs are involved in proteoglycans in cancer, proliferator-activated receptor (PPAR), AMPK, and AGE-RAGE signaling pathways, and ECM–receptor interaction (Table S9). The PPAR signaling pathway, ECM–receptor interaction, and AMPK signaling pathway were associated with lipid metabolism.

### 3.4. Co-Expression Network of Protein-Coding Genes and LncRNAs

**Figure 5:**
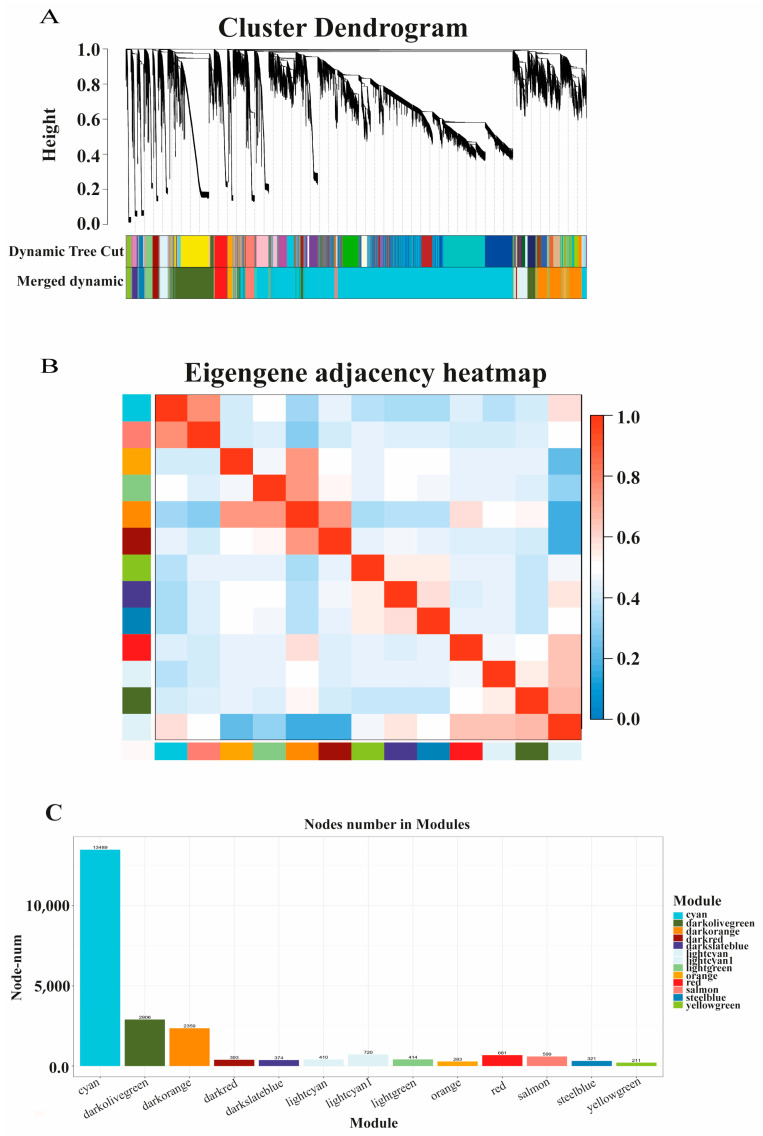
Weighted gene co-expression network analysis (WGCNA). (**A**) Hierarchical clustering dendrogram of lncRNA–mRNA co-expression modules. Each branches represents a cluster of lncRNAs or mRNAs. Dynamic tree cut represents original split module, and merged dynamic represents final merged modules. (**B**) Hierarchical clustering dendrogram of module eigengenes and heatmap of adjacencies using WGCNA; red and blue indicate positive and negative correlation, respectively. (**C**) Number of module genes; histogram represents numbers of lncRNAs or mRNAs in the co-expression module.

**Figure 6:**
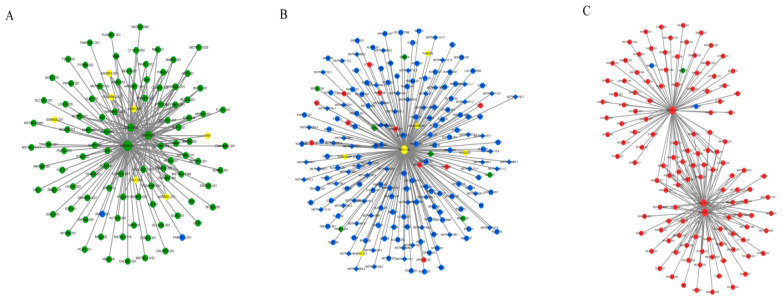
Co-expression network of mRNAs and lncRNAs in (**A**) molecular function module, (**B**) cellular component module, and (**C**) biological process module. Rhombic and circular nodes indicate mRNA and lncRNA, respectively. Color represents differential expression level: yellow: both upregulation and downregulation in development; red: upregulation: green: downregulation; blue: no changes.

A total of 19,398 protein-coding genes and 2491 lncRNAs were categorized into 13 modules. WGCNA showed that the molecular function, cellular component, and biological process modules were the three largest (gene number > 2000) among the 13 module profiles, accounting for 80.98% of the total genes (Figure 5A–C). *MED24*, *ANO6*, and *ZC4H2* were the hub genes in the molecular function module (Figure 6A); *TBPL1* was the hub gene in the cellular component module (Figure 6B); and *MOGS*, *ACADSB*, and *DNAJC25* were the hub genes in the biological process module (Figure 6C). The targeting relationships between mRNAs and lncRNAs in the same module are listed in Table 6. Functional enrichment analysis revealed that co-expression genes in the three largest modules were enriched in collagen biosynthetic and metabolic processes, GTPase activator activity, protein folding, carbohydrate biosynthetic process, acyl-CoA dehydrogenase activity, metabolic process, and ubiquitin-dependent protein binding (Tables S10–S12).

### 3.5. RT-qPCR Quantification of LncRNAs

**Figure 7:**
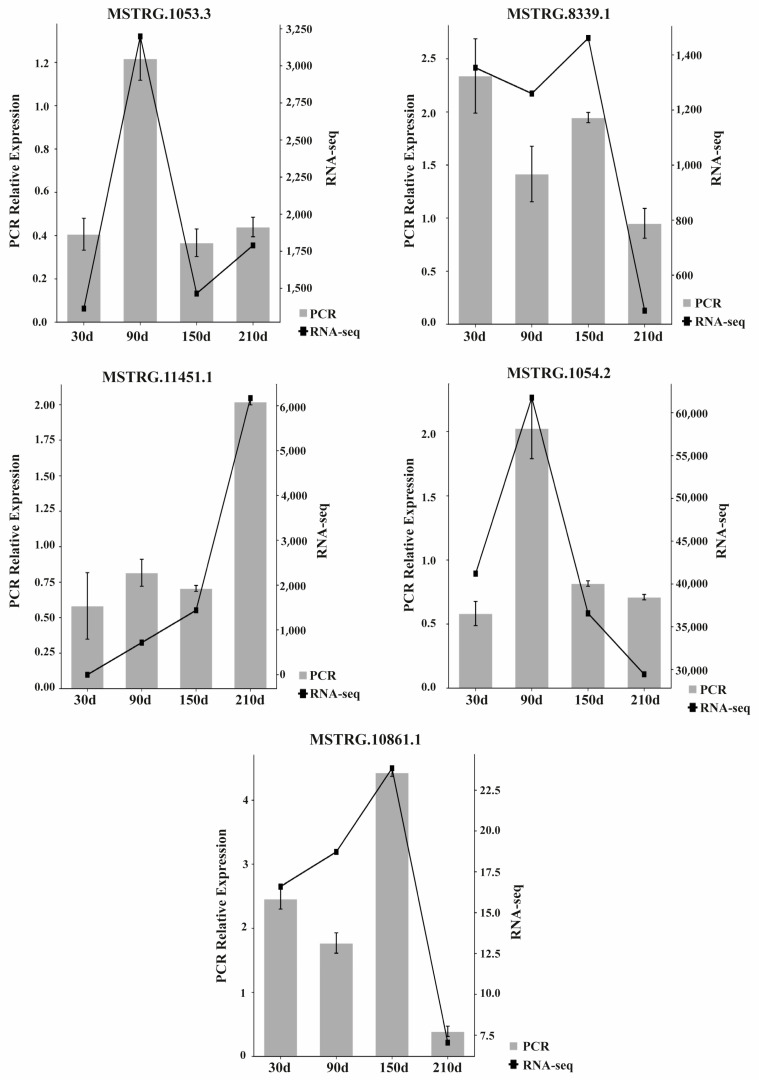
Transcription patterns of MSTRG.1053.3, MSTRG.11451.1, MSTRG.8339.1, MSTRG.10861.1, and MSTRG.1054.2 compared to expression patterns in the RNA-seq.

Five lncRNAs—MSTRG.1053.3, MSTRG.11451.1, MSTRG.8339.1, MSTRG.10861.1, and MSTRG.1054.2—were randomly selected from the novel lncRNAs and quantified by RT-qPCR at four development stages (30, 90, 150, and 210 days after birth). The results showed a concordance between the RNA-seq and RT-qPCR data, suggesting that the RNA-seq data was reliable (Figure 7).

## 4. Discussion

### 4.1. Differentially Expressed Protein-Coding Genes and lncRNAs

It has been widely recognized that lncRNAs play a significant role in tissue and organ development and metabolism regulation, and lncRNAs in the liver may influence digestion and metabolism. In this study, many differentially expressed mRNAs (DEmRNAs) and differential expressed lncRNAs (DElncRNAs) were identified. In the DEmRNAs study, the greatest differences occurred at 30 days compared to the other stages. This phenomenon suggests that the expression profiles at 30 days may be critical in liver development. Variations in the dietary structure during the suckling and weaning periods may affect the digestive function, which may be correlated with different expressions of mRNAs [25,26]. However, most of the differentially expressed lncRNAs were downregulated at 210 days after birth, indicating that the regulatory role of lncRNAs at this stage is relatively poorer than at other stages. At that age, Ningxiang pig is almost sexually mature and has a mature body and a propensity to gain weight, which may be the reason why the differential expression of lncRNAs was downregulated at that stage.

Venn diagrams were constructed to classify the common DEmRNAs and DElncRNAs along the four stages of development. *ACSL3*, *CES1*, *CYP2C42*, *CYP4A24*, and *PLIN4* were associated with fatty acid metabolism in common DEmRNAs. In the common DElncRNAs, MSTRG.31876.3 and MSTRG.33238.1 showed the highest expression levels at 150 days after birth. MSTRG.31876.3 was predicted to *PCF11* and *AGO1*, which are primarily involved in transcriptional regulation; and MSTRG.33238.1 was predicted to several target genes: *CDHR5*, *CYP4A24*, *HSD17B3*, and *TICAM1*, among which *CYP4A24* encoded for cytochrome p450. *CYP4A24*, also known as fatty acid omega-hydroxylase, regulates omega- and (omega-1)-hydroxylation of various fatty acids [27,28]. *TICAM1*, encoding for TLR domain-containing adapter molecule 1, actively participates in innate immunity against invasive pathogens to protect the host [29]. The findings from this study suggest that DEmRNAs and DElncRNAs may be associated with lipid metabolism, which may affect the meat quality of fatty pig breeds, including Ningxiang pigs. Research on the function of detected DEmRNAs and DElncRNAs should be carried out in order to gain deeper knowledge of the role and regulation of these DEmRNAs and DElncRNAs. A deeper understanding of these genes may help producers to manipulate or enhance the production of pigs. 

### 4.2. Time-Series Analysis of Protein-Coding Genes and LncRNAs

STEM analysis found that there was a dynamic and similar pattern of expression between mRNAs and lncRNAs during the development process in the liver. STEM analysis in this study revealed that mRNA profiles 21, 24, and 25 belonged to the same cluster, which was enriched for sterol and steroid biosynthesis and transport, triglyceride catabolism, lipoprotein particles, and cholesterol transport and esterification. In profile 21, *APOA1*, encodes for apolipoprotein A1 as part of the high-density lipoprotein (HDL) used to transport fat molecules back to the liver for excretion, had higher expression levels at 90, 150, and 210 days compared to 30 days after birth. *APOA1* has been reported to be regulated by *APOA1-AS*, an antisense lncRNA, as a negative transcriptional regulator [30]. We also found that *APOA4*, *APOM*, and *ABCG* were involved in the transport of lipoprotein particles and cholesterol. In addition, the KEGG pathway study showed that these genes were predominantly involved in lipid-related pathways, such as arachidonic acid metabolism, fatty acid biosynthesis, fatty acid metabolism, and PPAR signaling pathway. 

The genes in mRNA profile 4 were mainly enriched for fibril organization, vasculogenesis, GTPase activator activity, and regulation of kinase activity. The lncRNAs in profile 4 were primarily involved in long-chain fatty acids, membrane rafts, angiogenesis, and identical protein binding. Angiogenesis was also seen to have a key role in tumor growth and metastasis. A previous study reported that when the liver is damaged, regeneration of the liver is associated with angiogenesis, or is at least partially dependent on angiogenesis [31]. Target gene *Vezf1* of lncRNA in lncRNA profile 4 was found to encode the zinc finger transcription factor, which is transcribed in the endothelial cells during angiogenesis development and regulation, suggesting that *Vezf1* may promote liver development and tissue repair [32]. In addition, target gene *CPT1B*, encoding carnitine palmitoyltransferase 1B, a member of the carnitine acetyltransferase family, functions in regulating long-chain fatty acid beta-oxidation by transporting long-chain fatty acyl-CoAs from cytoplasm to mitochondria [33]. The expression of lncRNA encoded genes was higher at 30 days than 90, 150, and 210 days after birth, coinciding with mRNA transcription, which indicates that the gene may play a significant role during the suckling period in meeting the energy requirements of Ningxiang piglets.

*CAV1*, *PACSIN2*, and *Cdc42* in a particular mRNA profile 4 were exactly the target genes of lncRNAs in lncRNA profile 4. A previous study found that *CAV1* encoded for caveolin-1 protein, a scaffolding protein in caveolar membranes, which is primarily involved in endocytosis [34], cell transport, cell signal transduction and regeneration [35], intracellular cholesterol homeostasis [36], and lipid metabolism [37], which may influence liver diseases [38]. Various receptors, channels, and signal transduction mechanisms perform their biological role in caveolae where *CAV1* and *PACSIN2* interact. *PACSIN2* encodes the protein kinase C and casein kinase substrate mediating the shape formation of caveolae in plasma membranes, suggesting that *PACSIN2* is a fundamental protein involved in cellular homeostasis and disease via caveolae regulation [39]. *CDC42*, encoding a small GTPase-associated plasma membrane and identified as the Rho subfamily, participates in cellular responses, epithelial cell polarization processes [40], cell migration, positive regulation of cytokinesis [41], regulation of mitotic nuclear division, and GTPase activity [42]. Functional analysis of mRNAs and lncRNAs in this study further verified their biological role in lipid, saccharide, and protein metabolism. Meanwhile, KEGG pathway analysis of lncRNAs in profile 4 suggested that lncRNAs are primarily involved in the PPAR signaling pathway, ECM–receptor interaction, and AMPK signaling pathway associated with lipid metabolism, indicating that lncRNAs are actively involved in the lipid metabolism regulation in the liver of Ningxiang piglets 30 days after birth. These results suggest that the lipid metabolism is essential during the early development of piglets, and the liver plays a significant role in regulating the lipid metabolism. The lipid metabolism is crucial in maintaining normal homeostasis in piglets, especially during early development. An ideal lipid metabolism may enhance the growth and survival of piglets along the growing period.

### 4.3. Co-Expression Network of Protein-Coding Genes and LncRNAs

In the lncRNA–mRNA co-expression network, *MED24*, *ANO6*, and *ZC4H2* were identified as hub genes in the molecular function module. *MED24*, encoding mediator complex subunit 24 (TRAP100), a transcriptional coactivator complex involved in the induced transcription of almost all genes dependent on RNA polymerase II [18], has several biological functions associated with the lipid metabolism pathway by the proliferator-activated receptor-α (PPAR-α) and thyroid hormone signaling pathways [43]. In the cellular component module, *TBPL1* was classified as a hub gene. *TBPL1*, encoding TATA box-binding protein-like 1, also known as TLF and TRF2, belongs to the TATA binding protein (TBP) paralog despite lacking the ability to bind TATA elements and interact with transcription factor II A and B, mediating the transcription of most ribosomal proteins to regulate gene transcription in development and differentiation. 

*MOGS*, *ACADSB*, and *DNAJC25* were the hub genes in the biological process module. *MOGS* encode mannosyl-oligosaccharide glucosidase isoform 1, which removes the distal α1,2-linked glucose residues from Glc(3)-Man(9)-GlcNAc(2) oligosaccharide precursor. *MOGS* is substantially involved in glucosidase activity, mannosyl-oligosaccharide glucosidase activity, oligosaccharide metabolic process, and protein N-linked glysosylation [22]. A previous study showed that MOGS protein deficiency or *MOGS* mutation could lead to congenital glycosylation disorders (CDGs), which could lead to retarded development, low immunoglobulin levels, and abnormal liver function [44]. *ACADSB* encodes a short/branched chain specific acyl-CoA dehydrogenase (SBCAD), known as 2-methylbutyryl-CoA dehydrogenase (MBD), which catalyzes the first step of L-2-methylate short acyl-CoA compounds in the mitochondria [23]. *ACADSB* also functions in inisoleucine catabolism, fatty acid β-oxidation, and lipid metabolism [24]. *DNAJC25* encodes DNAJC homologous subfamily C member 25, known as HSP40 homolog, which binds to chaperon HSP70 by its J domain to facilitate ATP hydrolysis for protein folding, unfolding, translation, and degradation, and had particularly higher expression in the liver than adjacent normal tissues [25]. 

During the development of piglets, the liver is often injured by many factors, including weaning stress and digestive tract diseases [45]. Increased transcription of *HSP70* has been reported to reduce liver injury and promote liver health during the weaning period [46]. *HSP40* may interact with *HSP70* and they may function together in a variety of physiological processes, suggesting that *HSP40/DNAJC25* could play an important role in liver health, particularly when under stress or injured. This obviously shows that the co-expression network of protein coding genes and lncRNAs is significantly involved in liver protection. The liver is important in maintaining the heath and growth of an animal. If the liver is injured, the homeostasis will be harmed. We can observe that liver protection is important during the early stage of development, which might be due to the incomplete development of the immune system. It is necessary to protect the liver in order to ensure the survival of piglets. 

## 5. Conclusions

In conclusion, the present study reveals the expression profiles and functional networks on mRNAs and lncRNAs in the liver of Ningxiang piglets across four developmental stages (30, 90, 150, and 210 days after birth). We found that most of the DEmRNAs were upregulated at 30 days, but most were downregulated at 210 days. A complex interaction between mRNAs and lncRNAs was identified, indicating that lncRNAs may be a critical regulatory element in mRNAs according to STEM and WGCNA analysis. mRNAs and lncRNAs have similar patterns of expression in module profile 4, indicating that they have functional and regulatory relationships. Module profile 4 is related to fibril organization, vasculogenesis, GTPase activator activity, and regulation of kinase activity. We must point out that *CAV1*, *PACSIN2*, and *CDC42* in the particular mRNA profile 4 were only the target genes of lncRNAs in that profile, which suggests a possible regulatory relationship between lncRNAs and mRNAs. The expression of these genes and lncRNAs in profile 4 was the highest at 30 days, indicating that these RNAs may play a critical role during the suckling period to meet the dietary requirements of piglets. The findings of the current study lay a foundation for the study of lncRNAs in Ningxiang pigs and offer new insights for lncRNA functions in the liver at various stages of development. Research on the function of detected DEmRNAs and DElncRNAs should be carried out in order to gain deeper knowledge of the role and regulation of these DEmRNAs and DElncRNAs. A deeper understanding of these genes may help producers to manipulate or enhance the production of pigs.

## Figures and Tables

**Table 1:** Forward and reverse primers used for gene quantification by RT-qPCR.

Name	Sequence (5’ to 3’)
MSTRG.1053.3-F	ACTTGGGAAGAAAGCAATTTTAAGA
MSTRG.1053.3-R	TGTAGTCCCAGCTACTCGGG
MSTRG.11451.1-F	AGACATCCGAGCCTGGGATA
MSTRG.11451.1-R	CGTTTCAGAAAGCGTTGGAAGT
MSTRG.8339.1-F	GGCATATGGAGGTTCCCAGG
MSTRG.8339.1-R	GCGCAGTGGTTAACGAATCC
MSTRG.10861.1-F	GAGCCTGATTCCTCCAGCTC
MSTRG.10861.1-R	CCAGCCACAGCAATCAGAGA
MSTRG.1054.2-F	TGTAGTCCCAGCTACTCGGG
MSTRG.1054.2-R	ACAGGGTCTCGCTATGTTGC

**Table 2:** Statistics of raw and clean reads after quality control.

Sample	Raw Reads	Raw Bases	Clean Reads	Clean Bases	Error Rate (%)	Q20 (%)	Q30 (%)	GC Content (%)
30d-1	110,376,466	16,666,846,366	108,613,548	14,971,657,073	0.0241	98.25	95.24	55.15
30d-2	113,435,178	17,128,711,878	111,741,648	15,445,970,217	0.0241	98.27	95.16	54.05
30d-3	115,191,290	17,393,884,790	113,234,706	15,440,805,570	0.0242	98.22	95.10	54.40
90d-1	111,744,756	16,873,458,156	110,334,062	14,899,645,712	0.0237	98.48	95.60	51.03
90d-2	112,938,114	17,053,655,214	111,212,336	15,058,781,243	0.0237	98.47	95.59	52.15
90d-3	108,509,032	16,384,863,832	107,067,364	14,561,278,014	0.0237	98.48	95.53	51.38
150d-1	92,416,686	13,954,919,586	90,822,938	12,377,703,789	0.0239	98.38	95.32	50.39
150d-2	92,911,502	14,029,636,802	91,797,388	12,720,267,538	0.0238	98.44	95.40	49.73
150d-3	84,857,708	12,813,513,908	83,747,750	11,702,176,288	0.0241	98.34	95.16	49.99
210d-1	103,579,630	15,640,524,130	102,245,470	14,008,181,654	0.0237	98.51	95.54	49.84
210d-2	101,318,164	15,299,042,764	99,445,274	13,643,601,019	0.0237	98.45	95.52	52.12
210d-3	106,488,022	16,079,691,322	104,848,812	14,255,190,000	0.0237	98.45	95.56	50.78

**Table 3:** Statistics of mapping to reference genome.

Sample	Clean Reads	Mapped Reads	Mapping Rate (%)
30d-1	108,613,548	100,520,492	92.55
30d-2	111,741,648	103,608,606	92.72
30d-3	113,234,706	104,826,456	92.57
90d-1	110,334,062	104,211,698	94.45
90d-2	111,212,336	105,230,225	94.62
90d-3	107,067,364	101,424,972	94.73
150d-1	90,822,938	85,849,715	94.52
150d-2	91,797,388	85,907,029	93.58
150d-3	83,747,750	78,654,116	93.92
210d-1	102,245,470	95,722,450	93.62
210d-2	99,445,274	92,960,760	93.48
210d-3	104,848,812	98,178,592	93.64

**Table 4:** Differential expression of mRNAs.

Groups	Total DEmRNAs	Upregulated	Downregulated
30 vs. 90 d	7345	3473	3872
30 vs. 150 d	7971	4039	3932
30 vs. 210 d	7634	3761	3873
90 vs. 150 d	2309	1434	873
90 vs. 210 d	2754	1518	1236
150 vs. 210 d	1717	479	1238

**Table 5:** Differential expression of lncRNAs.

Groups	Total DElncRNAs	Upregulated	Downregulated
30 vs. 90 d	734	646	88
30 vs. 150 d	600	521	79
30 vs. 210 d	456	195	261
90 vs. 150 d	135	64	71
90 vs. 210 d	671	29	642
150 vs. 210 d	576	24	552

**Table 6:** Hub genes (target genes of lncRNAs) and associated lncRNAs in the same module.

mRNA	Module	Function of mRNA	Associated lncRNAs
***MED24***	Molecular function	Interact with RNA polymerase II to promote formation of transcriptional pre-initiation complex to induce gene expression [17]	MSTRG.2158.2 MSTRG.34993.2 MSTRG.16183.1 MSTRG.17517.2
***ANO6***	Molecular function	Essential component for calcium-dependent exposure of phosphatidylserine on cell surface, essential for triggering clotting system and deposition in bone mineralization [18]	MSTRG.31383.3 MSTRG.9728.2 MSTRG.33542.1 MSTRG.25050.1 MSTRG.40598.2 MSTRG.18175.1 MSTRG.42141.2 MSTRG.860.1 MSTRG.24048.3 MSTRG.13083.1
***ZC4H2***	Molecular function	ZC4H2 may improve channel activity and turnover of plasma membrane and is identified as potential candidate for X-linked mental retardation [19]	MSTRG.33542.1 MSTRG.40598.2 MSTRG.11929.4 MSTRG.3259.4
***TBPL1***	Cellular component	TBPL1 may play an important role in transcription by RNA polymerase II as a component of the transcription factor complex [20]	None
***MOGS***	Biological process	MOGS may cleave distal alpha-1,2-linked glucose residue from Glc(3)-Man(9)-GlcNAc(2) oligosaccharide precursor [21]	MSTRG.3283.1
***ACADSB***	Biological process	Catalyze dehydrogenation of short branched chain acyl-CoA derivatives in metabolism of fatty acids [22,23]	MSTRG.6256.1 MSTRG.22802.1 MSTRG.11451.1 MSTRG.24004.1 MSTRG.26481.9 MSTRG.32831.1 MSTRG.29185.1 MSTRG.3444.1
***DNAJC25***	Biological process	DNAJC25 may play an important role in cell protection, protein folding, refolding, aggregation and degradation, and protein translocation [24]	MSTRG.3827.1 MSTRG.8329.3 MSTRG.20562.1

## Data Availability

The datasets used and analyzed in the current study are available from the corresponding author on reasonable request.

## Supplementary Materials

The following are available online at https://www.mdpi.com/article/10.3390/biology10040310/s1, Table S1: Identified novel lncRNAs in liver. Table S2: Total differentially expressed mRNAs in liver. Table S3: Total differentially expressed lnvRNAs in liver. Table S4: Common DEmRNAs. Table S5: Enriched gene ontology functions of common DEmRNAs. Table S6: GO enrichment in mRNA module profile 4 of STEM. Table S7: GO enrichment in lnvRNAs module profile 4 of STEM. Table S8: KEGG enrichment in mRNAs module profile 4 in STEM. Table S9: KEGG enrichment in lncRNA module profile 4 of STEM. Table S10: GO enrichment in molecular function module of WGCNA. Table S11: GO enrichment in cellular component module in WGCNA. Table S12: GO enrichment in biological module of WGCNA.
