# Supplementary material for: Insight into Liver lncRNA and mRNA Profiling at Four Developmental Stages in Ningxiang Pig
Source: Biology (Basel). 2021 Apr 8;10(4):310. doi: 10.3390/biology10040310 (PMC8068270; doi:10.3390/biology10040310)
Supplement: Supplementary file 1 [file biology-10-00310-s001.zip › supplement information/Figure S1.docx]

Figure S1. Sample clustering of WGCNA analysis.
